# Understanding the background and clinical significance of the WHO, WOAH, and EMA classifications of antimicrobials to mitigate antimicrobial resistance

**DOI:** 10.3389/fvets.2023.1153048

**Published:** 2023-03-17

**Authors:** Ronette Gehring, Jonathan P. Mochel, Ivo Schmerold

**Affiliations:** ^1^Department Population Health Sciences, Faculty of Veterinary Medicine, Institute for Risk Assessment Sciences (IRAS), One Health Pharmacology, Utrecht University, Utrecht, Netherlands; ^2^Department of Biomedical Sciences, Iowa State University College of Veterinary, Medicine, Ames, IA, United States; ^3^European Federation for Pharmaceutical Sciences (EUFEPS), Mannheim, Germany

**Keywords:** WHO, WOAH/OIE, EMA, veterinary medicine, antimicrobials, classification

## Abstract

In Europe, the classification systems of the WHO, WOAH (founded as OIE), and EMA are the prevailing standard documents guiding the prudent use of antibiotic substances. While the WHO document “Critically important antimicrobials for human medicine” eponymously focusses on the use in humans, the other two documents, “OIE List of Antimicrobial Agents of Veterinary Importance” and “EMA Categorization of antibiotics for use in animals,” concentrate exclusively on the prudent use of antibiotics in animals. One common purpose of these classification systems is to provide guidance in making sound decisions on the choice of antibiotics for treating humans as well as animals. Although the latest editions of these compendia refer to one another and bear a clear resemblance at the category levels, some of the substances are grouped into unequal classes. This review illustrates the specific perspectives of the three categorization systems under consideration. The arguments raised for different classifications between the WHO and the EMA are exemplified for amoxicillins without beta-lactamase inhibitors, macrolides, sulfonamides, and colistin. For the daily clinical use of antibiotics, veterinarians should consider the EMA document, and, under tentative circumstances, consult the OIE list.

## 1. Introduction

Internationally, several classification systems have been developed for antibiotics with the goal of optimizing effective treatments for infectious diseases while mitigating and controlling the emergence and spread of antimicrobial resistance (AMR). Among the various approaches used to categorize antimicrobials, the most recognized systems in European countries are those of the World Health Organization (WHO), the World Organization for Animal Health (WOAH; founded as OIE)^1^ and the European Medicines Agency (EMA) (1–4). These classifications all aim to guide medical doctors and veterinarians in making sound decisions regarding antibiotic treatments of humans and animals, respectively. However, these systems differ in their methodologies, which has led to divergent classifications of some antimicrobial classes or substances. This divergence can be confusing for veterinarians in practice, especially since they must consider many animal species and a wide spectrum of specific pathogens.

The present review summarizes and explains the main differences between the WHO, WOAH, and EMA classification systems, with a special focus on the information that is relevant for veterinarians in practice. The terms “antimicrobial” and “antibiotic” are used interchangeably, as in the different international documents cited in this manuscript. Usually, antimicrobials comprise not only antibiotics but also antivirals, antifungals and antiprotozoals.

## 2. Classification of antimicrobials by the World Health Organization

The WHO's list of “*critically important antimicrobials for human medicine*” (WHO CIA List) was originally developed by following recommendations from two consecutive expert meetings organized by the Food and Agriculture Organization of the United Nations (FAO), the WOAH and the WHO. It was updated in 2007, 2009, 2011, 2013, 2016, and most recently in 2019 [the current version; (1)]. Since its establishment, several changes have been made to the list.

The WHO CIA List (1) focuses on the clinical requirements of human medicine and divides antibiotics into three main categories: (1) “*critically important antimicrobials (CIA)*,” (2) “*highly important antimicrobials (HIA)*,” and (3) “*important antimicrobials (IA)*.”

This comparatively extensive document shows an annotated table with antibiotics used both in human and veterinary medicine, thus emphasizing the interrelationship between both disciplines. The common use of identical antibiotic substances or classes of antibiotics is a source of concern. Although the WHO classification is focused on antibiotics important for human health, it explicitly accounts for the transfer of antimicrobial resistance genes from animals to humans.

The current sixth revision of the WHO document subdivides the CIA category into “*high priority critically important antimicrobials*” and “*highest priority critically important antimicrobials*.”

### 2.1. WHO criteria used for the classification of antimicrobial substances

Two main criteria (C1 and C2) are used for the WHO classification of antimicrobials into CIA, HIA and IA. A specific classification for antimicrobials used (solely) in veterinary medicine is not included in the WHO-CIA document (1):

C1: “*The antimicrobial class is the sole, or the one of limited available therapies, to treat serious bacterial infections in people*.” C2: “*The antimicrobial class is used to treat infections in people caused by either: (1) bacteria that may be transmitted to humans from non-human sources, or (2) bacteria that may acquire resistance genes from non-human sources*” (e.g., animals, water, foodstuff, or the environment).

### 2.2. WHO categories

With these two main criteria, the WHO defines three main categories of antimicrobials.

*Critically Important Antimicrobials (CIA):* This category comprises classes fulfilling both criteria C1 and C2. In addition, three prioritization factors are applied (P1, P2, and P3) for the subdivision of the CIA category into two subgroups. In brief, prioritization factor P1 refers to antimicrobial classes used to treat a large number of patients with infections for which only limited antimicrobials are available, P2 refers to the frequency of use in human medicine or in certain high-risk groups, and P3 refers to classes used to treat human infections “*for which extensive evidence exists on the transmission of resistant bacteria or genes from non-human sources*.”

CIA fulfilling all 3 prioritization criteria (P1–P3) are designated as “*highest priority critically important antimicrobials*” (Highest Priority CIA). Classes not fulfilling all these criteria are grouped together as “*high priority critically important antimicrobials*” (High Priority CIA).

Among the highest priority CIA are the following:

- Quinolones/fluoroquinolone (e.g., danofloxacin, difloxacin, enrofloxacin, ibafloxacin, marbofloxacin, orbifloxacin, and pradofloxacin),
- Macrolides and ketolides (tulathromycin, erythromycin, spiramycin, tylosin, tilmicosin, tildipirosin, tylvalosin, and gamithromycin),
- 3rd/4th generation cephalosporins (cefoperazone, ceftiofur, cefovecin, and cefquinome),
- Polymyxins (colistin, polymyxin B), and
- Glycopeptides.

With the exception of glycopeptides, all of these classes are licensed for use in animals in the European Union (EU).

*Highly important antimicrobials (HIAs)*: Antimicrobial class meets either C1 or C2 criteria.

Many classes of the HIA category are authorized for use in veterinary medicine, e.g., amphenicols, cephalosporines (1st and 2nd generations), penicillins, sulfonamides, and tetracyclines.

*Important antimicrobials (IA):* Antimicrobial class complying with none of the two criteria C1 and C2.

Among the IAs are, for example, nitroimidazoles and nitrofurans (both classes not licensed for food-producing animal species) and pleuromutilins.

## 3. Classification of antimicrobials of the World Organization for Animal Health

The “*OIE List of Antimicrobial Agents of Veterinary Importance”* was revised in 2018; over the course of this revision, its terminology was adapted to that of the WHO-categorization scheme (2). In contrast to the WHO and EMA classifications, only substances for use in food-producing animals are considered by the WOAH system. No substances solely licensed for use in humans and none used (in some countries) as performance enhancers are included. In its classification document, the WOAH distinguishes 10 different target animal species in which the respective antibiotic compounds are used. This approach makes the list relatively complex for some classes with varying compound-specific classifications or indications (e.g., polypeptides, and quinolones/fluoroquinolones).

It is worth mentioning that the OIE list is based on a survey from 2005 taken from member states of the WOAH and international organizations. The response to this survey was analyzed, and eventually, a list of antibiotic agents of veterinary importance was produced that was endorsed by the Biological Standards Commission. This list has repeatedly been revised and updated since then and was finally adopted in May 2018 by the Assembly of WOAH Delegates. Following a technical review, the list was endorsed by the Scientific Commission in February 2019 (2).

### 3.1. WOAH criteria for the classification of antimicrobial substances

Two criteria form the basis for categorizing veterinary antibiotics into three classes:

Criterion 1 (response rate): This criterion was met when the majority of the respondents (more than 50%) confirmed the importance of a class of antimicrobials.

Criterion 2 (treatment of serious disease): The criterion was fulfilled when substances of this class were “*(…) identified as essential against specific infections and there was a lack of sufficient therapeutic alternatives*.”

### 3.2. The WOAH categories

*Veterinary Critically Important Antimicrobial Agents (VCIA):* Both criteria 1 and 2 are fulfilled (e.g., tetracyclines, amphenicols, macrolides, and ketolides; Table 1).

**Table 1:** Classification, indications, and target species of antibiotic classes by the WHO, WOAH and EMA [without EMA Cat. A (Avoid) antibiotics].

**Antibiotic class (EMA terminology)**	**WHO**	Indication/comments (WHO)^*^	**WOAH**	**Target species (WOAH)**	**Indication/comments (WOAH)**	**EMA (Cat. D)**
Tetracyclines (chlortetracycline, doxycycline, oxytetracylin, tetracycline)	HIA	Limited therapy; infections due to *Brucella* spp., *Chlamydia* spp., and *Rickettsia* spp. Different resistance mechanisms (e.g., minocycline, doxycycline, chlortetracycline), against some bacteria (e.g., *Acinetobacter*).	VCIA	API, AVI, BOV, CAM, CAP, EQU, LEP, OVI, PIS, SUI	Very important for treating many bacterial and chlamydial diseases in a broad range of animal species. No alternatives in the treatment against heartwater (*Ehrlichia ruminantium*) and anaplasmosis (*Anaplasma marginale*).	D
Aminopenicillins, without beta-lactamase inhibitors (amoxycillin, ampicillin, metampicillin)	High priority CIA	Limited therapy for Listeria and *Enterococcus* spp. May result from transmission of *Enterococcus* spp., Enterobacteriaceae, including *Escherichia coli*, from non-human sources.	VCIA	AVI, BOV, CAM, CAP, EQU, LEP, OVI, PIS, SUI	Penicillins are used in the treatment of septicaemias, respiratory and urinary tract infections. Very important, penicillins treat many diseases in a broad range of animal species. Few economical alternatives.	D
Natural, narrow-spectrum penicillins (beta lactamase-sensitive penicillins) (benzathine benzylpenicillin, penethamate hydriodide, phenoxymethylpenicillin)	HIA	In certain geographic settings, the class may be one of limited therapies for streptococcal infections, yaws and syphilis. May result from transmission of penicillin-resistant *Staphylococcus aureus*, from non-human sources.				D
Anti-staphylococcal penicillins (beta-lactamase-resistant penicillins)		The class may be one of limited therapies for staphylococcal infections (*S. aureus*). May result from transmission of *S. aureus*, including MRSA, from non-human sources.				
Aminoglycosides: spectinomycin only	IA	In some areas, spectinomycin may be one of the limited antimicrobials still active against *Gonococcus*.May result from transmission of Enterobacteriaceae, including *E. coli*, from non-human sources; there is no demonstrated transmission from *E. coli* to *Gonococcus*.	VCIA	AVI, BOV, CAP, EQU, LEP, OVI, PIS, SUI	Used for respiratory infections in cattle and enteric infections in multiple species.	D
Sulfonamides, dihydrofolate reductase inhibitors and combinations (formosulfathiazole, sulfadiazine, sulfadoxine, trimethoprim)	HIA	In certain geographic settings the class may be one of limited therapies for acute bacterial meningitis, systemic non-typhoidal *Salmonella* spp. infections, and other infections. Transmission of Enterobacteriaceae (e.g., *E. coli*) from non-human sources.	VCIA (Sulfonamides, Diaminopyrimidine, combinations)	AVI, BOV, CAP, EQU, LEP, OVI, PIS, SUI	Extremely important; wide range of applications and the nature of the diseases; bacterial, coccidial and protozoal infections; wide range of animal species.	D
Cyclic polypeptides (bacitracin); (colistin and poymyxin B see Cat. B)	IA	High numbers of people affected by diseases in health care facilities in many countries for which the antimicrobial is the sole or one of few therapies available. In multiple countries, there is high use in people in critical care settings or where multidrug resistant organisms are prevalent. Colistin-resistant bacteria and the *mcr* family genes can be transmitted *via* the food chain.	VHIA	AVI, BOV, CAP, EQU, LEP, OVI, SUI	Bacitracin is used against necrotic enteritis in poultry where available. Indication in septicaemias, colibacillosis, salmonellosis, and urinary infections. Widely used against gram-negative digestive infections.	D
Steroid antibacterials (fusidic acid)	HIA	One of few limited therapies for infections with MRSA. May result from transmission of MRSA from non-human sources.	VIA	BOV, EQU	Fusidic acid is used in the treatment of ophthalmic diseases in cattle and horses.	D
Nitroimidazoles (metronidazole)	IA	This class may be one of the limited therapies for anaerobic infections including *Clostridium difficile*.	Not mentioned	–	–	D
Nitrofuran derivatives (furaltadone, furazolidone)	IA	–	Not mentioned	–	–	D
**Antibiotic class (EMA terminology)**	**WHO**	Indication/comments (WHO)^*^	**WOAH**	**Target species (WOAH)**	**Indication/comments (WOAH)**	**EMA (Cat. C)**
Aminoglycosides (except spectinomycin) (amikacin, apramycin, dihydrostreptomycin, gentamicin, neomycin)	High Priority CIA	Sole or limited therapy as part of treatment of enterococcal endocarditis and MDR tuberculosis and MDR Enterobacteriaceae. In some countries, there is a high proportion of use in patients in health care settings with serious infections. High frequency of use in human medicine. Transmission of *Enterococcus* spp., Enterobacteriaceae (including *E. coli*), and *Mycobacterium* spp. from non-human sources.	VCIA (2-deoxystreptamine)	API, AVI, BOV, CAP, EQU, LEP, OVI, PIS, SUI	Extremely important; important in treating septicaemias; digestive, respiratory and urinary diseases. Gentamicin is indicated for *Pseudomonas aeruginosa* infections with few alternatives.	C (except spectinomycin = D)
Aminopenicillins, in combination with beta lactamase inhibitors (amoxycillin + clavulanic acid; ampicillin + sulbactam)	High Priority CIA	In certain geographic settings, there may be a high absolute number of people affected by diseases for which these antimicrobials are the sole or one of few therapies available. High frequency of use. Limited therapy for *Listeria* and *Enterococcus* spp., may result from transmission of *Enterococcus* spp., Enterobacteriaceae, including *E. coli* from non-human sources.	VCIA	AVI, BOV, CAP, EQU, OVI, PIS, SUI	Wide range of applications, extremely important; treatment of septicaemias, respiratory and urinary tract infections, few economical alternatives available.	C
Cephalosporins, 1st- and 2nd-generation, and cephamycins (cefacetrile, cefadroxil, cefalexin)	HIA	May result from transmission of Enterobacteriaceae (*E. coli*) from non-human sources.	VHIA	BOV, CAP, EQU, OVI, SUI	Cephalosporins are used in the treatment of septicaemias, respiratory infections, and mastitis.	C
Amphenicols (florfenicol, thiamphenicol; chloramphenicol is banned for use in food animals)	HIA	In certain geographic settings, the class may represent one of the limited therapies for acute bacterial meningitis, typhoid and non-typhoid fever, and respiratory infections. May result from transmission of Enterobacteriaceae, including *E. coli* and *Salmonella* spp., from non-human sources.	VCIA	AVI, BOV, CAP, EQU, LEP, OVI, PIS, SUI	Extremely important (wide range of applications and the nature of the diseases treated). Particular importance in treating some fish diseases (no or very few treatment alternatives). Useful alternative in respiratory infections of cattle, swine and poultry. Florfenicol is used to treat pasteurellosis in cattle and pigs.	C
Lincosamides (clindamycin, lincomycin, pirlimycin)	HIA	May result from transmission of *Enterococcus* spp. and *Staphylococcus aureus*, including MRSA, from non-human sources.	VHIA	API, AVI, BOV, CAP, OVI, PIS, SUI, BOV, SUI, AVI	Essential in the treatment of Mycoplasmal pneumonia, infectious arthritis and haemorrhagic enteritis of pigs.	C
Pleuromutilins (tiamulin, valnemulin)	IA	Thus far only used as topical therapy (retapamulin). To date, no transmission of resistance in *S. aureus*, including MRSA, from non-human sources.	VHIA	AVI, CAP, LEP, OVI, SUI	Essential against respiratory infections in pigs and poultry. Also essential against swine dysentery (*Brachyspira hyodysenteriae*).	C
Macrolides and ketolides (erythromycin, gamithromycin, tulathromycin, tylosin, tylvalosin)	High priority CIA	High absolute number of people affected by diseases for which these antimicrobials are the sole or one of few therapies available. High frequency of use in human medicine. Transmission of resistant *Campylobacter* spp. from non-human sources.	VCIA	API, AVI, BOV, CAP, EQU, LEP, OVI, PIS, SUI	Extremely important; wide range of applications. Used to treat Mycoplasma infections in pigs and poultry, haemorrhagic digestive disease in pigs (*Lawsonia intracellularis*) and liver abscesses (*Fusobacterium necrophorum*) in cattle, where they have very few alternatives. Additionally, used for respiratory infections in cattle.	C
			VIA (only avilamycin)	AVI, LEP, SUI	Avilamycin is used for enteric diseases of poultry, swine and rabbit. This class is currently only used in animals.	C
Rifamycins: only rifaximin	High priority CIA	Limited therapy as part of treatment of mycobacterial diseases (tuberculosis); single drug therapy may select for resistance. May result from transmission of *Mycobacterium* spp. from non-human sources and MDR *Staphylococcus aureus* through the food chain.	VHIA (Rifampicin, Rifaximin)	BOV, CAP, EQU, LEP, OVI, SUI	Authorized only in a few countries and with a very limited number of indications (mastitis) and few alternatives.Rifampicin is essential in the treatment of *Rhodococcus equi* infections in foals.	C
**Antibiotic class (EMA terminology)**	**WHO**	**Indication/comments (WHO)** ^*^	**WOAH**	**Target species (WOAH)**	**Indication (WOAH)**	**EMA (Cat. B)**
Cephalosporins, 3rd- and 4th-generation (with the exception of combinations with beta-lactamase inhibitors) (cefoperazone, cefquinome, ceftiofur; cefovecin only pet animals)	Highest priority CIA	Limited therapy for acute enterobacterial meningitis and disease due to *Salmonella* ssp. in children, infections due to MDR Enterobacteriaceae (worldwide), treatment of neutropenic patients with persistent fever. Transmission of Enterobacteriaceae (*E. coli, Salmonella* spp.) from non-human sources.	VCIA	BOV, CAP, EQU, OVI, LEP, SUI, AVI	Wide range of indications, extremely important; treatment of septicaemias, respiratory infections, mastitis; limited alternatives.	B
Polymyxins (colistin, polymyxin B)	Highest priority CIA (Colistin)	Limited therapy for infections MDR Enterobacteriaceae (*Klebsiella* spp., *E. coli, Acinetobacter, Pseudomonas* spp.). Transmission of Enterobacteriaceae from non-human sources.	VHIA (polymxin B, colistin)	BOV, CAP, EQU, OVI, LEP, SUI, AVI	Treatment of septicaemias, colibacillose, salmonellosis, urinary infections.Colistin: gram-negative enteric infections.	B
Quinolones: fluoro and other quinolones (danofloxacin, enrofloxacin, flumequine, marbofloxacin; ibafloxacin, pradofloxacin only pet animals)	Highest priority CIA	Limited therapy for *Campylobacter* spp., invasive disease due to *Salmonella* spp., MDR *Shigella* spp. infections. Transmission of *Campylobacter* spp. from non-human sources.	VHIA (first gen.)	BOV, CAP, EQU, OVI, LEP, SUI, AVI	First. gen.: Nalidixic acid, oxolinic acid, flumequine: Treatment of septicaemias, infections (colibacillosis).	B
Quinolones: fluoro and other quinolones (danofloxacin, enrofloxacin, flumequine, marbofloxacin; ibafloxacin, pradofloxacin only pet animals)	Highest priority CIA	Limited therapy for *Campylobacter* spp., invasive disease due to *Salmonella* spp., MDR *Shigella* spp. infections. Transmission of *Campylobacter* spp. from non-human sources.	VCIA (second gen.)	BOV, CAP, EQU, OVI LEP, SUI, AVI	Second Gen.: Wide range of applications, extremely important, treatment of septicaemias, respiratory and enteric diseases.	B

* Classification, indications/comments and target species are taken from WHO (1), WOAH (2), and EMA, [(4), infographic], respectively (abridged text, with modifications); only EMA categories D, C, B are considered; EMA Category A (Avoid) substances are not authorized as veterinary medicines in the EU. Additionally, glycopeptides, lipoglycopeptides, glycylglycines, lipopeptides, monobactams, oxazolidinones, antipseudomonal penicillins, fosfomycin, drugs used solely to treat tuberculosis or other mycobacterial diseases, pseudomonic acid, streptogramins, sulfones, tetracyclines 5th gen., nitrofurans, and nitroimidazoles, as mentioned in the WHO document, are not registered in the EU for use in animals.

API, bee; AVI, avian; BOV, bovine; CAP, caprine; CAM, camel; EQU, equine; LEP, rabbit; OVI, ovine; PIS, fish; and SUI, swine; VCIA, veterinary critically important antimicrobial; VHIA, veterinary highly important antimicrobial; VIA, veterinary important antimicrobial; CIA, critically important antimicrobial; HIA, highly important antimicrobial; IA, important antimicrobial; B, restrict; C, caution; D, prudence; MRSA, methicillin-resistant *Staphylococcus aureus*; MDR, multidrug resistant.

*Veterinary Highly Important Antimicrobial Agents (VHIA):* One of the criteria is met, criterion 1 or 2 (e.g., cephalosporines, 1st and 2nd generation and cephamycins, lincosamides; Table 1).

*Veterinary Important Antimicrobial Agents (VIA):* Neither of the two criteria are met (e.g., steroid antimicrobials; Table 1).

The WOAH list does not provide subcategories for the VCIA class the same way as the WHO does for CIA antibiotics. Nevertheless, some of the substances listed as VCIA are categorized as being of the highest priority in the WHO list (fluoroquinolones, cephalosporines 3rd/4th gen). It is accepted that while it is important that substances of the VCIA category remain available to veterinary medicine to ensure animal health, they also need to be used in a highly responsible manner (2). This concern also applies to colistin, which was categorized as VHIA in the OIE list and as Highest Priority CIA in the WHO document (1).

## 4. Classification of antimicrobials by the European Medicines Agency

The EMA's Antimicrobial Advice Ad Hoc Expert Group (AMEG), a group established to support EMA's Committee for Medicinal Products for Veterinary Use (CVMP) and the Committee for Medicinal Products for Human Use (CHMP), has categorized antibiotics according to their potential risk of increased AMR for public health when used in animals as well as the need for their use in veterinary medicine (5). Veterinarians are encouraged to verify the AMEG categorization before prescribing any antibiotic for animals in their care. As explicitly stated, the EMA/AMEG categorization does not replace other official (national) guidelines on the use of antimicrobials. Additionally, other provisions, such as the supporting information given in the Summary of Product Characteristics for available medicines, mandatory constraints regarding treatment of food-producing species, information on regional variations in diseases, antibiotic resistance, and national prescribing policies remain valid.

### 4.1. The EMA categories

In its recently published document “*EMA categorization of antibiotics for use in animals for prudent and responsible use*,” the EMA distinguishes among four categories from antimicrobials for use in food production as well as pet animals, which are designated categories A, B, C, and D [Table 1; (4)].

*Category A (Avoid):* Antibiotics of this category are not approved for use in animals in the EU and are banned from use in food-producing animals but can be applied to companion animals, e.g., in cases of a therapeutic gap by way of exception [Article 112, (6)].

Category A compounds include amdinopenicillins, carbapenems, monobactams, oxazolidinones, glycopeptides, and other antibiotic classes of high importance in human medicine. For all these classes no veterinary medicinal products are on the market.

It should be mentioned at this point that the new regulation (6) has a provision for “*(…) strengthening of the prudent use of antimicrobials by restricting the use of substances in animals which are of critical importance for preventing or treating life-threating infections in humans*.” Antimicrobials or groups of antimicrobials reserved for the treatment of specific infections in humans will be designated by the Commission by means of implementing acts [Art. 37 (6), p. 72]. This list as well as the criteria of the reservation of antibiotics for use in humans include high importance to human health, risk of transmission of resistance, and non-essential need for animal health (7–9).

Understandably, these compounds might, at least initially, not be included in Category A.

*Category B (Restrict):* Category B antibiotics comprise compounds listed under CIA in the WHO document (e.g., polymyxins, quinolones, fluoroquinolones and other quinolones; Table 1).

No further differentiation is made in the EMA document for this category of antimicrobials, as is performed by the WHO for the CIA category.

These antimicrobials should only be used carefully to minimize public health risks. They should be considered for administration to animals “*(…) only when there are no antibiotics in Categories C or D that could be clinically effective*.” Their “*(…) use should be based on antimicrobial susceptibility testing, where possible*.”

*Category C (Caution):* For antibiotics of this category, there are alternatives in human medicine, but there are no alternatives in veterinary medicine for some indications belonging to Category D. Category C antibiotics “*(…) should only be considered when there are no antibiotics in Category D that could be clinically effective*” (e.g., amphenicols, macrolides, pleuromutilins; Table 1).

*Category D (Prudence):* Category D antibiotics should be used as first-line treatments whenever possible. They should be used prudently and “*(…) only when medically needed*” (e.g., aminopenicillins, without beta-lactamase inhibitors, tetracyclines, nitroimidazoles; Table 1).

All antibiotics listed in these categories may not be used unnecessarily, not for overly long treatment periods and should not be underdosed. Detailed rules applying to prudent use can be found in Commission Notice 2015/C 299/04 (9).

## 5. Discussion

Strict observation of antimicrobial classification systems is a critical component of the prudent use of antibiotics. Many other considerations influence the choice of an adequate antimicrobial candidate, such as the results of antimicrobial testing, the target animal species, the route of administration, animal husbandry practices, and a clinical context demanding either prophylactic, metaphylactic or therapeutic use of the medication, be it in individual animals or whole flocks. These issues are usually addressed in the respective specific product information or regional provisions.

Evidently, a number of antimicrobials are placed in different categories by the WHO, WOAH, and EMA. While some divergence can be expected given the slightly different goals of these classifications, there are clearly major differences in the classification of some antimicrobials.

All antibiotic classes listed in EMA Category B (Restrict) are classified by the WHO as CIA and by the WOAH as VCIA or VHIA. However, there are also antimicrobials categorized by the EMA as Category D (Prudence) that are grouped into the WOAH category VCIA and in the WHO category CIA. Some EMA Category D antibiotics are classified as HIA by the WHO, but none are classified as category IA (Table 1). In principle, the AMEG widened its focus to the risks of antimicrobials in human medicine and the spread of resistance from animals to humans, whereas the WOAH classification is based on a survey among WOAH member states for the antibiotics that are essential for animal health.

Among the “*OIE list of antimicrobials of veterinary importance*” that are considered critical for animal health, there are compounds that are also listed as critically important for human health (e.g., fluoroquinolones, 3rd- and 4th-generation cephalosporins and colistin) (2). The WOAH recommendations state that these antimicrobials should not be used for prevention or as a first-line treatment and that their use should be based on the results of microbiological culture and antimicrobial susceptibility testing.

Here, aminopenicillins without beta-lactamase inhibitors, macrolides, sulfonamides, and polymyxins (colistin) may serve as sample antibiotics for a brief illustration of the logic underlying these different classifications. Additional details regarding the classifications and indications of antimicrobial substances can be found in Table 1.

### 5.1. Aminopenicillins without beta-lactamase inhibitors

One noticeable difference in the current versions of the WHO and EMA documents pertains to the group of aminopenicillins without beta-lactamase inhibitor (e.g., amoxicillin, ampicillin) classified as high priority CIA by WHO and included in Category D by EMA, i.e., a category defining veterinary first-line antibiotics of the lowest risk group.

The rationale for this apparent disconnect lies in the specific considerations regarding indications, clinical importance and risks of resistance selection related to the use of this antibiotic class in human and veterinary medicine. Aminopenicillins are important for treatment of a wide spectrum of infections in various animal species including companion animals. Resistance to aminopenicillins is widespread in both humans and animals, with different types of transferable resistance genes and mechanisms being involved. Although aminopenicillin selects for resistance to beta-lactam antibiotics, it was concluded that veterinary use is unlikely to contribute greatly to additional resistance development in humans. These antibiotics were therefore included in EMA Category D and not in a higher category based on their clinical importance in veterinary medicine (4, 10).

In contrast, the antibiotics in Category C pose a higher risk of AMR development in humans through their veterinary use (4). Therefore, “*(…) these antibiotics should only be used when there is no available substance in Category D that would be clinically effective*.” The principles of prudent use relate to all antimicrobials regardless of category.

### 5.2. Macrolides

The WHO categorized macrolides and ketolides as HPCIA, while the EMA put macrolides for veterinary use in group C (Caution; Table 1).

EMA Category C (Caution) lists substances that are included in different WHO categories. “*For those substances proposed for inclusion in this category, there are in general alternatives in human medicine in the EU but there are few alternatives in veterinary medicine for certain indications. Antibiotic classes that may select for resistance to a substance in Category A through specific multi resistance genes have also been placed in this category*” (4). As assessed by the AMEG, antibiotics placed in Category C have a higher risk of AMR development in humans and animals than Category D antibiotics (4). Category C (Caution) antibiotics should only be used when there are no clinically effective alternatives in Category D (4).

### 5.3. Sulfonamides

Sulfonamides, including those used in combination with dihydrofolate reductase inhibitors (e.g., trimethoprim), are used in human medicine to treat Enterobacteriaceae and staphylococci infections (e.g., MRSA). They are classified as HIA by the WHO but are included in Category D in the EMA system (11). The categorization of compounds in D is explained by a lower risk of AMR when compared to Category C and, therefore, these category members are recommended as a first-line antibiotic treatment. According to AMEG Category D, these antibiotics are not selected for resistance to Category A substances. Another justification is the existence of alternative treatments in human and veterinary medicine for their respective fields of application (Table 1).

### 5.4. Colistin (polymyxin E), highly ranked both in human and veterinary medicine

Colistin (polymyxin E) is grouped in EMA Category B (restrict) and as HPCIA by the WHO, i.e., its use is highly restricted in both systems. Colistin, a polymyxin antibiotic, is only nationally authorized in the EU for administration to animals. However, since the 1950s, it has been widely used for oral (group) treatment of gastrointestinal tract infections in pigs, poultry, cattle, small ruminants, and rabbits caused by non-invasive *Escherichia coli* (12).

The EMA reported that between 2011 and 2021, polymyxin sales had declined by 79.5% in 25 EU countries (that provided data over that period). Polymyxins accounted for 2.2% of the total antimicrobial sales for food-producing animals in the 31 countries providing data to the European Surveillance of Veterinary Antimicrobial Consumption (ESVAC) project in 2018 (13–15).

In human medicine, colistin is one of the last resort antibiotics against multidrug-resistant gram-negative Enterobacteriaceae (enterobacterials*, Acinetobacter baumannii*, and *Pseudomonas aeruginosa*) (14). The specific clinical significance and use of colistin both in human and veterinary medicine is emphasized in special advice on the (restricted) use of colistin in animals (13). Colistin was categorized by the WHO as HPCIA because of its increasing use in the treatment of severe infections in humans and because of the detection of mcr genes both in isolates from humans and animals that conferred transmissible resistance to colistin and the spread of resistant bacteria along the food chain (1). In veterinary medicine, the use of colistin is, therefore, subject to regulations.

In this context it is worth mentioning that between 2017 to 2021 sales have dropped by 19.9% for 3rd- and 4th-generation cephalosporins, 8.5% for all quinolones, and 39.0% sales for polymyxins (all contained in EMA Category B; measured as mg/PCU). These data add up to a total sale reduction of 20.9% and refer to antibiotics for food-producing animals in the 31 countries providing data to the European Surveillance of Veterinary Antimicrobial Consumption (ESVAC) project (14).

## 6. Conclusions

Which classification scheme appears to be the most appropriate for everyday use by veterinary practitioners in the EU? The answer to this question is multifaceted, because all three classification systems described herein are functionally interconnected. Despite their common goal of ensuring effective treatment of bacterial infections while minimizing the development and spread of antibiotic resistance, there are inevitable differentiations in the perception of their clinical value and the associated risks.

In essence, the WHO classification of antimicrobials primarily focuses on the treatment of human patients. Their document is also meant to help regulators and stakeholders learn which antimicrobials are used in both humans and animals and to understand which class of antibiotics may pose a potentially higher risk of resistance to the human population from concomitant use in veterinary medicine. Therefore, the WHO document should be considered in conjunction with the “*OIE list of antimicrobials of veterinary importance*” (2) and the EMA document (4). However, the WHO document provides no practical information on the many and various clinical situations in veterinary medicine requiring antibiotic use.

The WOAH classification system contains antimicrobial agents only authorized for use in food-producing animals in WOAH member states, i.e., including states outside the European Union/European Economic Area (EU/EEA). Therefore, the list also encompasses antibiotic classes not permitted for use in food-producing animals in the EU/EEA (e.g., arsenicals, quinoxalines, and nosiheptide). The WOAH classification document provides specific data (e.g., indications, target species, and general remarks) of interest for veterinary practitioners. There is no further subdivision of antimicrobial classes in the VCIA group, and no specific information is provided on marketing authorisations granted by the different member states.

The EMA is responsible for the scientific evaluation, authorization and post-marketing monitoring of medicinal products licensed in the EU for both human and veterinary medicine. Consequently, the EMA document considers antibiotics for use in both food-producing and companion animal species. The EMA's AMEG was particularly involved in the conception of the EMA classification system and comprises specialists in human as well as veterinary medicine (16); the AMEG is responsible for considering the issues of AMR resistance and public health related to the use of veterinary medicinal products.

The EMA/AMEG classification of antimicrobials forms the basis for developing (regional) treatment protocols and the preparation of respective national guidelines for prescription practices under the “cascade” (outside the terms of the marketing authorisation), thus supporting the responsible use of antimicrobials (4). Notably, any local regulations on the use of antimicrobials remain valid. The EMA classification is based on and complies with the current pharmaceutical and veterinary drug regulation in force within the EU. Any future revision(s) of the classification is expected to comply with this regulation.

The EMA classification was conceived to serve the needs of veterinary practitioners, to support and facilitate the appropriate selection of an antimicrobial in their daily practice. The classification is straightforward and considers the clinical significance of antibiotics from their most restricted use (Category A) to substances indicated for use in first-line treatment scenarios (Category D).

Overall, the defined EMA categories are not in complete accordance with the systems of the WHO and WOAH. However, the latter two systems were considered for the preparation of the EMA categorization document. These facts make the EMA system appear as the first-line classification system to consult for antimicrobial treatments of animals.

## Author contributions

RG and JM together with IS initiated the overall concept of the manuscript. All authors provided passages with focus on veterinary issues and revised the temporary drafts and the final version of the manuscript.

## Footnotes

^1^Both abbreviations, WOAH and OIE, are used interchangeably.
